# A bibliometric analysis of the published road traffic injuries research in India, post-1990

**DOI:** 10.1186/s12961-018-0298-9

**Published:** 2018-03-01

**Authors:** Neeraj Sharma, Mohan Bairwa, B. Gowthamghosh, S. D. Gupta, D. K. Mangal

**Affiliations:** 1 Centre for Injury Research (CIR), The IIHMR University, Jaipur, India; 2 Johns Hopkins Bloomberg School of Public Health, Baltimore, United States of America; 3 Public Health and Epidemiology, The IIHMR University, 1, Prabhu Dayal Marg, Near Sanganer Airport, Jaipur, 302029 India

**Keywords:** Injury, Road crashes, Fatalities

## Abstract

**Background:**

Globally, road traffic injuries are the leading cause of death among those aged 15–29 years. However, road traffic injury research has not received adequate attention from the scientific community in low- and middle-income countries, including India. The present study aims to provide a bibliometric overview of research assessing road traffic injuries in India.

**Methods:**

We used Scopus to extract relevant research in road traffic injuries published from 1991 to 2017. This study presented the key bibliometric indicators such as trends of annual publications and citations, top 10 authors, journals, institutions and highly cited articles, citation analysis of articles, co-occurrence of keywords, etc. Analysis was performed using Scopus, Microsoft Excel, and VOS-viewer.

**Results:**

A total of 242 articles were retrieved with an h-index of 18, excluding self-citations. A steadfast growth of publications was documented in last decade, especially after the year 2010. The h-index of the top 10 authors, institutions, journals and highly cited articles did not surpass single digits. A network visualisation map showed that ‘traffic accident’, ‘male’, ‘adolescent’ and ‘child’ were the most commonly encountered key terms. The prominent authors were Gururaj G, Dandona R, and Hyder AA, whereas the top journals were the *Indian Journal of Forensic Medicine and Toxicology*, *Medico Legal Update*, and the *International Journal of Applied Engineering Research* and top institutions were the All India Institute of Medical Sciences, New Delhi, the Indian Institute of Technology, Delhi, and the Administrative Staff College of India.

**Conclusion:**

In India, road traffic injuries research is inadequate in quantity and quality, warranting greater attention from researchers and policy planners to address the burden of road traffic injuries.

## Background

In 1990, injuries in general ranked as the 12th contributor in global disease burden in terms of disability adjusted life years (DALY), rising to 7th rank in 2010 and projected to reach 5th by the year 2030 [1, 2]. Road traffic injuries (RTIs) account for approximately 30% of injury-related deaths worldwide, and are the most common cause of death among people aged 15–29 years [3]. In India, approximately 28% of total DALYs lost due to injuries are attributed to RTIs alone [4]. In 2016, there were 150,785 deaths occurring in 480,652 road crashes. Further, RTI-related deaths have increased by 43% over the last 10 years [5]. Unless new initiatives and intense efforts are made, the total number of road traffic deaths in India is likely to surpass 250,000 by 2025 [6].

In India, RTIs, particularly those involving pedestrians, cyclists and motorised 2- or 3-wheelers, are a significant cause of preventable death, predominantly in men of productive age [7]. Research in RTIs has not received much attention from the scientific community due to a lack of focus in national policies and funding opportunities, especially in low- and middle-income countries (LMIC). In India, such as in most LMICs, the research output on key public health issues, including RTIs, remains poor. Indeed, only 4.4% of the total health research studies published in India during 2002 were related to public health sciences [8] and the contribution of injury research (2001–2008) was only 0.7% (RTI research, 0.1%), which is grossly inadequate to address the current disease burden [9].

National Crime Record Bureau data shows a clearly rising burden of RTI-related morbidity and mortality in India [9]. Realising the urgency, the Government of India initiated a drive to address the increasing RTI burden in the 11th Five Year Plan (2007–2012) by developing a pan-India network of trauma care facilities to provide immediate treatment for accident victims along the Golden Quadrilateral Corridor as well as the North-South and East-West Corridors. The objective of this scheme was to reduce the number of preventable deaths because of road accidents to 10% [10]. Nevertheless, there is no published research of appropriate quality on the assessment of RTI burden or on the effectiveness of interventions [8]. Responding to these issues, the Indian Council of Medical Research (ICMR) has recently invited countrywide research proposals on RTIs specifically focusing on surveillance, health system preparedness, and barriers and facilitators during the golden hour, as well as to develop interventions to address the problem of RTIs [11]. In addition, systematic reviews and bibliometric analyses may be valuable tools to fill this research gap. Bibliometric analyses may aid policy planners and researchers in identifying critical research areas for evidence-based decision-making and policy formulation in LMICs [12].

Therefore, the present bibliometric analysis aims to map RTI research output, assessing the quality of the research ecosystem in India, and identifying the focus areas and actors involved. This analysis will help inform researchers, academicians and the scientific community on the need and importance of research in this emerging public health issue.

## Methods

This study is a bibliometric analysis of research published in the field of RTIs in India. Bibliometrics, a widely used technique, involves the quantitative analysis of citations of published journal articles [12]. The bibliometric analysis methodology used herein followed that used in previous studies [8, 13, 14]. This study included the RTI research performed in India from 1990 to date.

### Search strategy

Several electronic databases may be used to perform bibliometric analyses to study research output. Herein, Scopus®, the largest global database of medical and social science research, was used since it is considered the most reliable and robust database of scientific literature. Scopus® has several advantages over other databases like Web of Science, EMABASE, PubMed, and Google Scholar [15], including a wide range of publications and more accurate data analysis.

In this study, we used the definition of RTI provided by WHO, namely “*a fatal or non-fatal injury incurred because of a collision on a public road involving at least one moving vehicle*” [16]. The keywords for developing the search strategy were determined using published review articles on RTIs and the WHO definition of RTI, in consultation with authors and subject experts. By using asterisks, we tried to retrieve all the possible keywords.

**Fig. 1:**
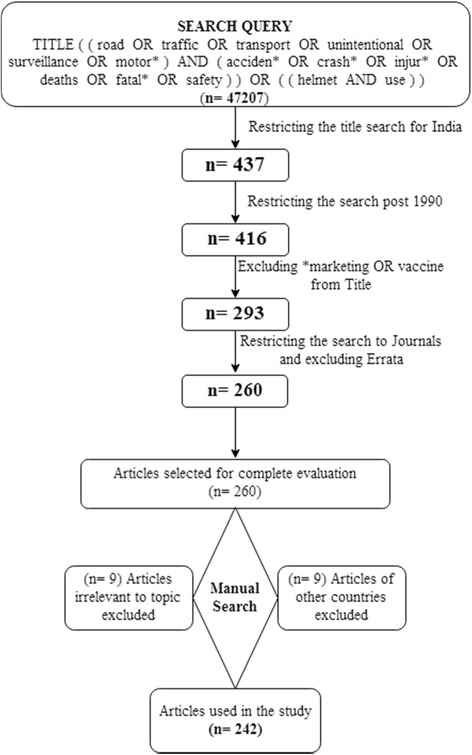
Search strategy

Using a title search only there was a strong possibility of false negative results. Therefore, we tried multiple search strategies, yet all of them led to a substantial number of false positive results without any significant inclusion of relevant articles. Thus, a title search query was performed, considering it avoided large numbers of false positive articles but would have missed only some of the true articles (Fig. 1).

### Inclusion and exclusion criteria

Studies published in journals (indexed in Scopus®) and either exclusively or in partly undertaken in India were included. We screened the title of all identified articles to ensure the reliability of the search and we reviewed the abstract in case of any confusion regarding the relevance of the article. In the case of papers without abstracts, we screened the full texts.

Books, book chapters, book series and errata documents were excluded using the refine and limit functionalities in Scopus® to restrict the analysis to literature in peer-reviewed journals. No language restrictions were made.

### Data analysis

We analysed annual trends of publications and their citations, the citation relationship among authors, citation analysis of articles, and the top 10 productive authors, journals and institutions and highly cited publications. The growth in the number of research publications and trends of citations over the years were presented using line diagrams. We calculated the Hirsh index (h-index) of authors, journals and institutions for RTI-related publications only. The h-index, introduced by JE Hirsch in 2005, is now widely accepted and employed as a measure of the total effective research output of a researcher, journal, institution or country. The h-index of a scientist has a value of x if x number of their published articles have each been cited at least x times in other papers, and their remaining articles have less than x citations each [17]. While calculating the h-index, we removed self-citations to improve the validity of search results.

The impact factor (IF) and SCImago Journal Rank (SJR) were presented for the top 10 journals. The IF is a measurement of visibility of articles in specific journals. It was originally developed by Eugene Garfield, and is calculated as the ratio of the number of citations in a given year divided by the number of ‘citable’ articles published in the previous two years. This information comes from the approximately 12,000 journals indexed by the Web of Science, which is published in the Journal Citation Report®. Citations, counted in the numerator, can be from any type of article from journals within the database, whereas only articles designated as research or review count in the denominator [18]. The SJR is a prestige metric based on the idea that ‘all citations are not created equal’. With SJR, the subject field, quality and reputation of the journal has a direct effect on the value of a citation [19].

We also performed citation analysis. VOSviewer is a software tool for constructing and visualising bibliometric networks to understand citation relationships [20]. Herein, we analysed journal co-citations, citation relationships between documents, co-occurrence of keywords, and co-authorship using density visualisation or networking maps using VOSviewer techniques [21].

Co-citation analysis is used to assess relatedness between two publications [22]. If one publication cites two publications, the two publications are treated as co-cited. The strength of the co-citation relationship between two publications depends on the largest number of publications by which the two publications are co-cited. In network visualisation maps, the thickness of the link between any two items is indicative of the strength of relationships, which is based on the number of lines between the two items, be they authors, institutions, journals, etc.

Manual analysis confirmed the validity of all the selected articles. Papers showing deviation from our objective of extracting relevant publications were excluded by using Boolean terms. Since it is also difficult to assess the quality of the research in bibliometric analysis, we used the total number of citations received, average number of citation received per article, h-index, IF and SJR as proxy measures of the quality of publications.

## Results

The Scopus® search retrieved 260 documents, all of which were manually reviewed and 18 documents were excluded. Of the 18 excluded documents, 9 were not relevant to RTIs and 9 were from countries other than India. Finally, we retrieved 242 documents for bibliometric analysis (Fig. 1). Of the 242 articles retrieved, the majority were original articles (*n* = 208, 85.95%), reviews (*n* = 16, 6.61%), editorials (*n* = 6, 2.48%), letters (*n* = 5, 2.07%), and others such as conference papers, short surveys and notes (*n* = 7, 2.89%). The retrieved documents were published in 108 peer-reviewed journals. The total number of citations for the 242 documents, at the time of data analysis (23 March 2017), was 1302 excluding self-citations by the authors. The average number of citations per article was 5.38. All the publications were available in English. The h-index of total retrieved articles was 18 (at least 18 documents were cited at least 18 times in peer-reviewed scientific publications).

### Annual distribution of publications and citations

**Fig. 2:**
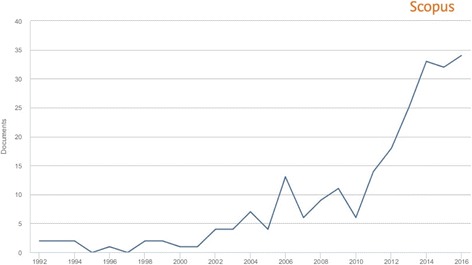
The total number of publications per year, 1992–2016

**Fig. 3:**
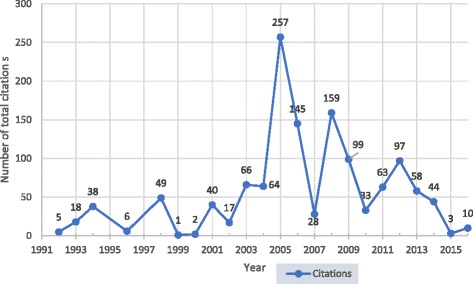
The total number of citations of articles related to RTIs in India published from 1991

The total number of publications on RTIs did not surpass single digits per year until 2005. However, a stead-fast growth in publications was documented over the last two and half decades (1991–2000: 12, 4.96%; 2001–2010: 65, 26.86%; 2011–2017: 165, 68.18%). An inverse relationship was observed in the total number of citations per year after 2005 (Figs. 2 and 3).

### Journals

A list of top 10 journals in the field of RTI research is shown in Table 1. The top 10 journals included approximately 36% of the total number of publications (*n* = 88, 36.67%). The *Indian Journal of Forensic Medicine and Toxicology* (*n* = 16, 6.67%), *Medico Legal Update* (*n* = 15, 6.25), and the *International Journal of Applied Engineering Research* (*n* = 11, 4.58%) were the top three journals, contributing more than 10 documents each. However, of the top 10 journals, only one had SJR > 1 (*Accident Prevention and Analysis*) and only four journals had an IF. Although all the top 10 journals are within the scope of forensic sciences and injury, no multidisciplinary journal including public health found a place in the list of top 10 journals.

**Table 1:** Top ten journals publishing on RTI research

SCR	Journal	Number of articles (A) (%)	Total no. of citations (C) (R)	C/A (R)	h-index (R)	IF (R)	SJR (R)
1	*Indian Journal of Forensic Medicine and Toxicology*	16	9	0.56	2	Nil	0.128 (8)
2	*Medico Legal Update*	15	21	1.4	3	Nil	0.110 (9)
3	*International Journal of Applied Engineering Research*	11	0	0	0	Nil	0.130 (7)
4	*Journal of Indian Academy of Forensic Medicine*	9	21	2.33	3	Nil	0.279 (5)
5	*Injury Prevention*	7	81	11.57	4	1.693 (2)	0.594 (3)
5	*International Journal of Injury Control and Safety Promotion*	7	12	1.71	2	0.888 (4)	0.294 (4)
7	*Journal of Forensic Medicine and Toxicology*	6	48	8	3	Nil	NA
7	*Medicine Science and the Law*	6	68	11.33	4	0.569 (5)	0.258 (6)
7	*Traffic Injury Prevention*	6	25	4.17	3	1.148 (3)	0.637 (2)
10	*Accident Analysis and Prevention*	5	279	55.8	4	2.070 (1)	1.1098 (1)

*SCR* standard completion ranking, *SJR* scientific journal rank, *IF* impact factor, *R* rank

**Fig. 4:**
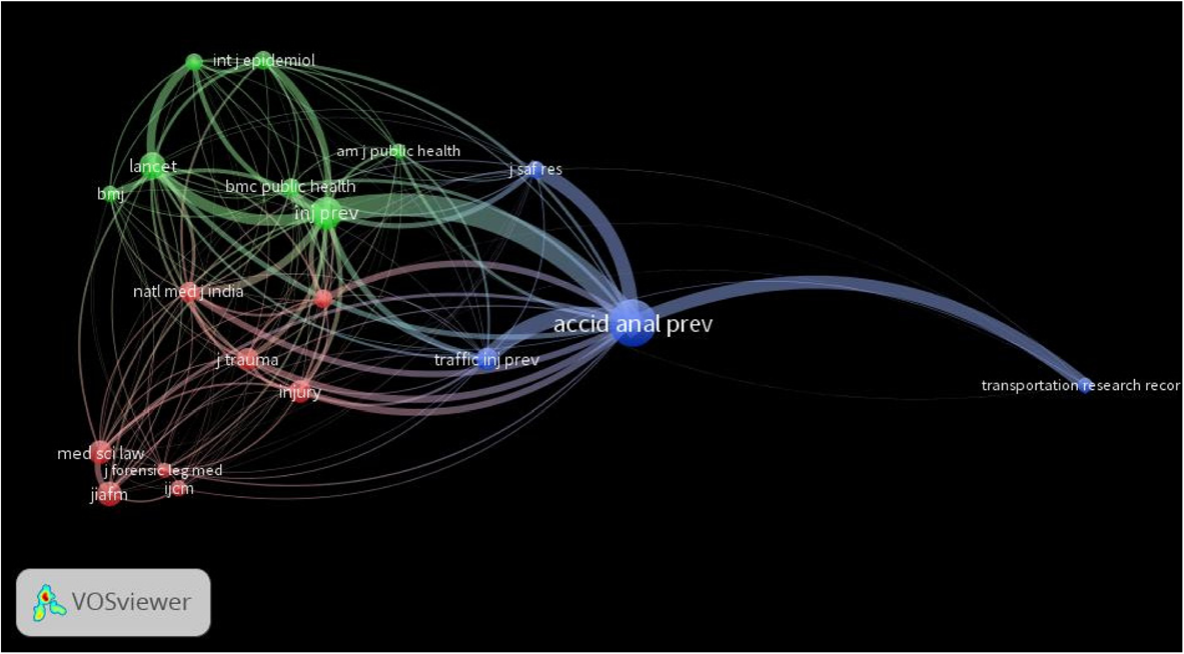
Journal co-citation analysis. Journals with a minimum of 20 citations were included in the network visualisation map to make the map readable and less crowded

Co-citation analysis of the most productive journals is depicted as a network visualisation map with a total of 3012 link strength (Fig. 4). *Accident Analysis and Prevention* had major links to *Injury Prevention* (link strength, 202), *Traffic Injury Prevention* (link strength, 141), *the Journal of Safety Research* (link strength, 125), and *Transportation Research Record* (link strength, 116). The circle size assigned for the journals correlates with the number of citations and the thickness of the net correlates with link strength.

### Institutions/Organisations

The top 10 productive institutions/organisations on RTI research in India are shown in Table 2. Of these, six institutions were governmental, three were private and one was an autonomous institution established by a public–private partnership. The All India Institute of Medical Sciences, New Delhi, was the most productive institution (*n* = 14), followed by the Indian Institute of Technology, Delhi (*n* = 11), and the Administrative Staff College (*n* = 10). The impact of the publications was assessed indirectly by the percentage contribution of highly cited articles from the top 10 institutions. The Administrative Staff College of India (15.2%) ranked ahead in the percentage of highly cited articles, followed by the Indian Institute of Technology Delhi (12.1%) and Johns Hopkins Bloomberg School of Public Health, Baltimore, United States of America (9.1%).

**Table 2:** List of top 10 institutions publishing on RTI research

SCR^a^	Institution	Number of documents	Total citations (R)	Citation/article (R)	HCA^b^	% HCA	h-index (R)^c^	Affiliated country^d^
1	All India Institute of Medical Sciences, New Delhi	14	88	6.3	2	6.1	5	India (Govt.)
2	Indian Institute of Technology, Delhi	11	96	8.7	4	12.1	4	India (Govt.)
3	Administrative Staff College of India	10	174	17.4	5	15.2	8	India (PPP)^e^
4	Postgraduate Institute of Medical Education and Research, Chandigarh	9	56	6.2	1	3.0	4	India (Govt.)
4	Johns Hopkins School of Public Health, Baltimore	9	81	9.0	3	9.1	5	USA (Private)
4	National Institute of Mental Health and Neuro-Sciences, Bangalore	9	92	10.2	1	3.0	4	India (Govt.)
7	Kasturba Medical College, Mangalore	7	64	9.1	2	6.1	6	India (Private)
8	Anna University, Chennai	6	1	0.2	0	0.0	1	India (Govt.)
9	Christian Medical College, Vellore	5	39	7.8	1	3.0	2	India (Private)
9	The University of Sydney, Sydney	5	43	8.6	0	0.0	4	Australia (Govt.)

^a^Equal institutions have same ranking number

^b^Articles with ≥ 15 citations were considered HCA; percentage of HCA was calculated by dividing total number of HCA by total number of publications for each institution/organisation assigned [33]

^c^Self-citations excluded

^d^In parenthesis finding status provided

^e^Public private partnership

*HCA* highly cited articles, *SCR* standard competition ranking

When we analysed research collaboration (network visualisation of co-authorship from different institutions), the All India Institute of Medical Sciences, a top contributor in RTI research in India, did not rank in the top 10 institutions (data not included).

### Authors

**Fig. 5:**
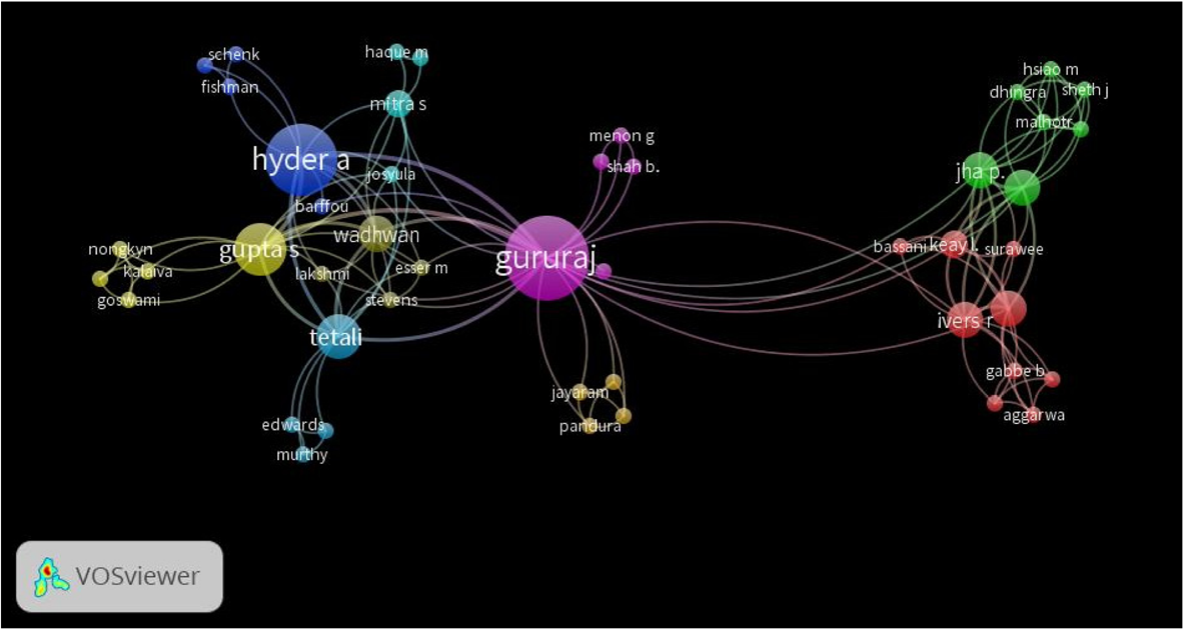
Co-authorship network visualisation map. All authors with co-authorship are included. Size of circle indicates total number of citations

The top 10 most prolific authors in the field of RTI research in India, with their affiliations and number of publications, are shown in Table 3. The list includes eight authors from India and two from the United States. Co-authorship among authors in RTI research in India, depicted using a network visualisation map (Fig. 5), included 47 authors with a 177 total link strength. Maximum collaboration was observed among Gururaj G, Hyder AA, Gupta S, Wadhwaniya S and Tetali S (link strength of 3–4 in each possible pair). The strength of collaboration is related to the thickness of connection between any two authors. Similar circle colours are considered as clusters where authors have close collaboration.

**Table 3:** Top 10 prolific authors publishing on RTI research

SCR^a^	Author	Number of published articles	Total citation (R)	Total citation/article (R)	h-index (R)	Affiliation (Institution, Country)
1	Gururaj G	10	91 (5)	9 (8)	4 (6)	National Institute of Mental Health and Neuro Sciences, India
2	Dandona R	8	154 (1)	19 (2)	7 (1)	Public Health Foundation of India, India
2	Hyder AA	8	81 (4)	10 (7)	5 (5)	Johns Hopkins Bloomberg School of Public Health, USA
2	Tiwari G	8	60 (9)	8 (9)	2 (9)	Indian Institute of Technology Delhi, India
5	Dandona L	7	135 (2)	19 (2)	6 (2)	Public Health Foundation of India, India
6	Kanchan T	6	63 (8)	11 (6)	6 (2)	All India Institute of Medical Sciences, New Delhi, India
6	Kumar GA	6	123 (3)	21 (1)	6 (2)	University of Texas at San Antonio, United States
6	Mohan D	6	70 (6)	12 (5)	3 (8)	Indian Institute of Technology, Delhi, India
9	Sagar S	5	12 (10)	2 (10)	2 (9)	All India Institute of Medical Sciences, New Delhi, India
10	Dogra TD	4	66 (7)	17 (4)	4 (6)	All India Institute of Medical Sciences, New Delhi, India

^a^Equal institutions have same ranking number

*SCR* standard competition ranking, *R* rank

### Highly cited articles

**Fig. 6:**
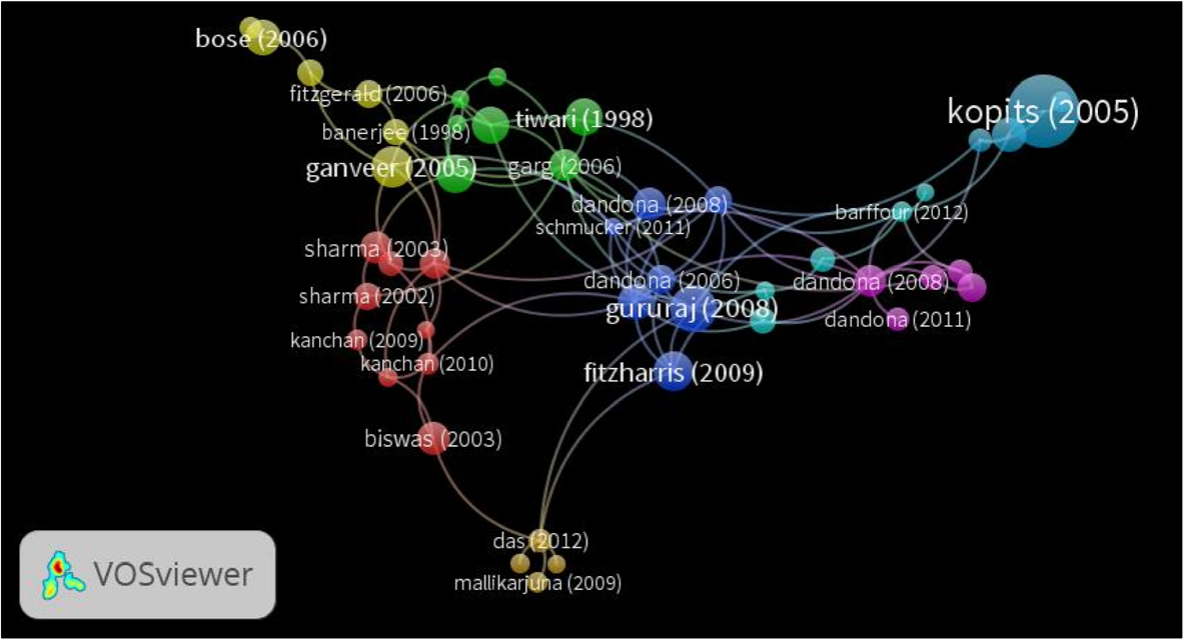
A network visualisation map of citation relationship between documents; 47 highly cited documents were included

The top 10 highly cited articles are shown in Table 4 [23–32]. The article by Kopits et al. [23], entitled “Traffic fatalities and economic growth”, achieved the highest number of citations (*n* = 203). Of the top 10 highly cited articles, three were published in the journal *Accident Analysis and Prevention*, while two were published in the *National Medical Journal of India*. Six studies were original research with a cross-sectional design, three secondary data analyses, and one was a review paper. The topics ranged from disease burden and injury characteristics and patterns to conflict analysis and relationship with development. The network visualisation map of citation relationships of highly cited documents (Fig. 6) includes eight clusters of 47 documents differentiated by various colours; these eight clusters have a total link strength of 98.

**Table 4:** Top 10 cited articles on RTI research (1996–2016)

SCR	Author (year of publication)	Title	Journal	Study design	Number of citations^a^
1	Kopits E (2005) [20]	Traffic fatalities and economic growth	*Accident Analysis and Prevention*	Secondary data analysis	200
2	Gururaj G (2008) [21]	Road traffic deaths, injuries and disabilities in India: Current scenario	*National Medical Journal of India*	Review	64
3	Ganveer GB (2005) [22]	Injury pattern among non-fatal road traffic accident cases: A cross-sectional study in central India	*Indian Journal of Medical Sciences*	Cross-sectional study	51
4	Fitzharris M (2009) [23]	Crash characteristics and patterns of injury among hospitalized motorized two-wheeled vehicle users in urban India	*BMC Public Health*	Cross-sectional study	43
5	Sharma BR (2001) [24]	Road-traffic accidents - A demographic and topographic analysis	*Medicine, Science, and the Law*	Cross-sectional study	40
6	Tiwari G (1998) [25]	Conflict analysis for prediction of fatal crash locations in mixed traffic streams	*Accident Analysis and Prevention*	Cross-sectional study	34
6	Sahdev P (1994) [26]	Road traffic fatalities in Delhi: Causes, injury patterns, and incidence of preventable deaths	*Accident Analysis and Prevention*	Cross-sectional study	38
7	Bose A (2006) [27]	Mortality rate and years of life lost from unintentional injury and suicide in South India	*Tropical Medicine and International Health*	Cross-sectional study	34
8	Dandona R (2004) [28]	Deaths due to road traffic crashes in Hyderabad city in India: Need for strengthening surveillance	*National Medical Journal of India*	Secondary data analysis	29
9	Garg N (2006) [29]	Exploring the relationship between development and road traffic injuries: A case study from India	*European Journal of Public Health*	Secondary data analysis	27

^a^Self-citations were excluded

*SCR* standard competition ranking

### Co-occurrence of all keywords

**Fig. 7:**
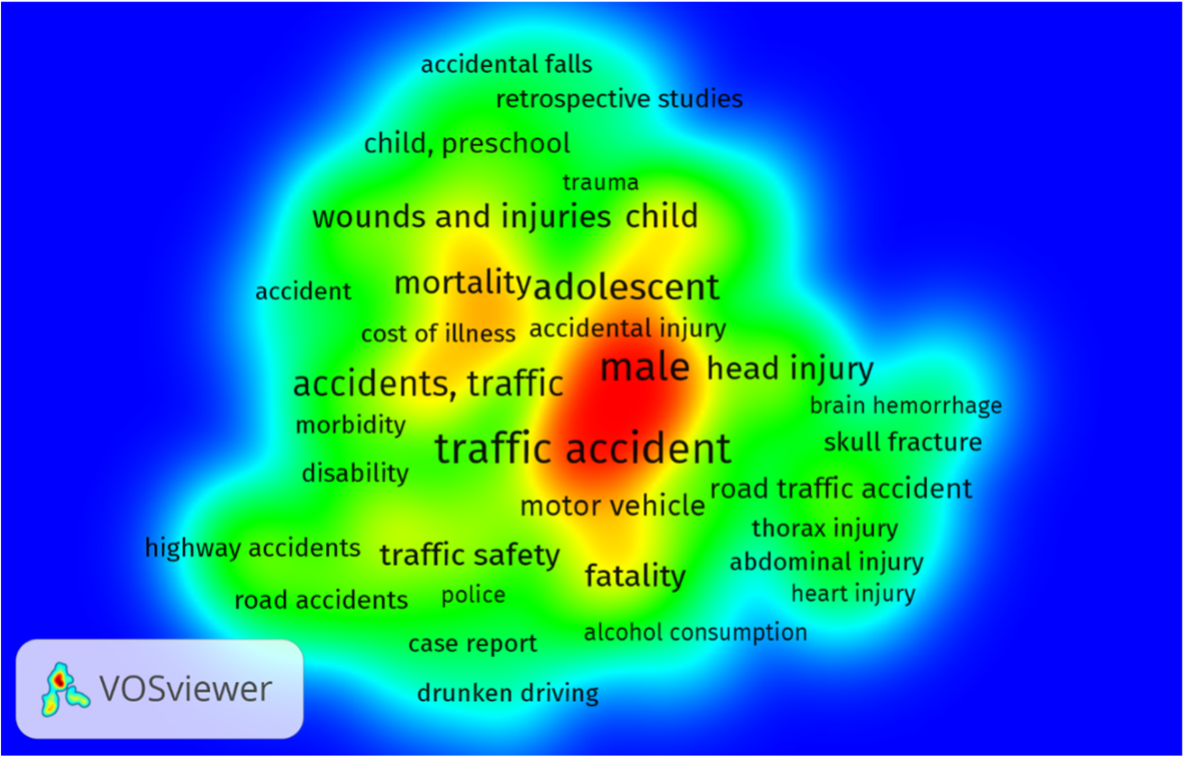
Density visualisation map of co-occurrence of all keywords

The density visualisation map of co-occurrence of all keywords included four clusters of 118 relevant keywords with a total link strength of 11,114 (Fig. 7). The density of a term reflects the number of related keywords in various documents in which both were found. The distance between two terms offers an approximate indication of the relatedness of the terms. The relatedness of terms was determined based on co-occurrences. Colours represent groups of terms that are strongly related to each other. In this map, we found the main keywords to be ‘traffic accident’, ‘male’, ‘adolescent’, ‘child, preschool’, ‘fatality’, etc. It indicated that research on ‘road safety’, ‘disability’ and ‘role of alcohol in crashes’ was largely ignored. The visualisation map also revealed that prominent keywords such as brain haemorrhage, skull fracture, thorax, and abdominal and heart injuries, which are primarily clinical entities, seemingly clustered distantly in a small segment on the map, suggesting that clinical research is conducted in isolation.

We also analysed the publications data by collaborative country based on author affiliations. The greatest amount of research activity contribution, in countries other than India (*n* = 41, 16.94%), was from the United States (*n* = 18, 7.44%), followed by Australia (*n* = 5, 2.07%) and Canada (*n* = 4, 1.65%). In India, the major contributing States in the field of RTI research were Karnataka (*n* = 34, 14.05%), Delhi and Tamil Nadu (*n* = 33; 13.64%).

## Discussion

In this study, we presented a bibliometric review of publications on RTIs in India. The analysis showed a steady quantitative increase in publications on RTI, especially after the year 2010, which reflected that this issue is receiving the attention of policy planners and researchers. This rise in publications might be due to a growth in the number of research journals, especially online journals, as well as the increased attention of the scientific community, networking and collaboration, and an increasing focus from agencies such as WHO, Bloomberg Philanthropies and the Government of India [3, 33–36].

We analysed the value of the h-index of retrieved articles, revealing an inadequate contribution and quality of research publications. We analysed the top 10 institutions, journals, authors and highly cited articles to identify the leading actors. This analysis provided insights on the quality of RTI research in India. The h-index of institutions based on RTI research in India also revealed low research productivity. None of the top 10 institutions achieved an h-index beyond single digits. Further, the study revealed that clinical research is being performed in isolation. Contribution to highly cited articles was no more than five articles by any institution, despite using a much lower cut off at 15 citations. Except for the Administrative Staff College, no institution in the top 10 had a total number of citations surpassing three digits.

Evaluation of the top 10 cited articles on RTI revealed that only five published articles were original articles with no more than 50 citations. Of the top 10 cited articles, none was multi-centric or multi-country or had an interventional design. No nationally representative study was included in the top 10 articles. This study reported that the top four journals did not have an IF; none of the top 10 journals had an h-index value of more than 4, and the SJR of the top 10 journals remained less than 1. Only one of the top 10 journals, *Accident Prevention and Analysis*, had an IF greater than 1. This finding is also in coherence with the analysis of the top 10 institutions in terms of quality. Thus, the study findings indicate an inadequate quality and amount of RTI research conducted in India, in agreement with previous analyses performed on the evaluation of public health research [8, 9, 37].

In India, the growth of the motor vehicle industry, liberalised economic policies of successive governments, aggressive media promotion for cars and bikes, increasing purchasing power, easy availability of loans and poor public transport facilities have possibly contributed in increasing motorisation and a changing transport scenario. Cyclist and motorcyclist transport is a major mode of travel in towns, rural areas and even most cities in India [7, 24, 38–40]. In such a scenario, there is an urgency of research and innovations in the field of RTIs, especially with regards to vulnerable road users, legislation and road behaviour, disability, etc. [3]. Herein, this lag is evident in the density visualisation map of all relevant keywords. Networking between the scientific community is quintessential to address this problem.

The Road Traffic Injuries Research Network, evolved at the Global Forum for Health Research in Geneva (1999), has raised significant interest among research collaborators and institutions in LMICs [35]. There is a need for creating such networks to promote research at the national and sub-national levels in India. Nevertheless, there has been increased attention in RTIs nationally and internationally, as evident from the launching of the pan-India trauma care network scheme [10], ICMR’s recent research initiative [11], the formation of Road Traffic Injuries Research Network and Road Safety collaboration [35], the declaration of “Road Safety decade (2011–2020)” [41], and the heavy fines for traffic rules violation in the recently amended Motor Vehicle (Amendment) Bill 2016 [42]. However, a comprehensive evidence base is needed to take informed actions on road safety, and prevention and management of RTI in India. This is a window of opportunity for India to break the downfall in the inverted U shaped loop (Kuznets phenomenon) RTI mortality pattern [32], as experienced in developed countries. Realising the need, the National Health Policy 2017 has highlighted the need for preventing RTIs and resulting deaths by developing appropriate intervention strategies. Our study findings will help inform the focus areas in developing a national research agenda to substantially reduce RTI burden and improve road safety.

This bibliometric analysis may be a significant guide for tracking the growth and quality of research in RTIs. To the best of our knowledge, there are few publications on bibliometric analysis in India to evaluate the research productivity of institutions, journals and key research and policy areas. Our study would open new opportunities to identify research areas in RTI and networking opportunities in India.

### Limitations

The present bibliometric analysis of RTIs might be biased due to the non-inclusion of publications in journals not indexed in Scopus®. This type of selection bias is not uncommon in studies based on published materials. Further, it is possible that we did not exhaust all possible keywords related to RTIs and accidents despite the rigorous search strategy employed in the analysis. In addition, we might have missed the inclusion of some publications given the cutoff of 1990 onwards, given that complete citations on the subject are available on Scopus® from 1996 onwards. Further, some authors had multiple affiliations, which may have affected the ranking of top institutions and could have appeared for more than one. Given these limitations, the study tried to provide the most recent information about all authors and publications, though the updating on Scopus® is a continuous process.

## Conclusions

RTI research output in India has increased significantly although the amount and quality of publications does not match the increasing burden of RTIs. Our study concluded that there are marked research inequities in relation to the RTI burden and geographic distribution, a lack of collaborative research between disciplines and networking among researchers, a lack of studies in risk factors and risk groups, and that clinical research is performed in isolation. Systematic priority setting, adequate funding and institutional capacity-building will be required to address the alarming situation of RTIs.
